# Perceived quality and willingness to continue using telemedicine services in patients with diabetes

**DOI:** 10.1093/eurpub/ckac129.569

**Published:** 2022-10-25

**Authors:** F Sanmarchi, E Maietti, L Palestini, D Golinelli, F Esposito, N Boccaforno, MP Fantini, P Di Bartolo

**Affiliations:** 1 Department of Biomedical and Neuromotor Sciences, Alma Mater Studiorum - Università di Bologna, Bologna, Italy; 2 Regional Agency for Health and Social Service, Regione Emilia-Romagna, Bologna, Italy; 3 Azienda Unità Sanitaria Locale della Romagna, Rimini, Italy; 4 AUSL Diabetes Unit Romagna, Ravenna, Italy

## Abstract

**Background:**

Plenty of literature reported the applicability and usefulness of telemedicine and teleassistance (TMTA) services in the management of diabetes and other chronic conditions. Specifically, TMTA proved to be effective for conditions that require radical lifestyle modifications, tailored pharmacological interventions, and periodic monitoring of clinical health status. The purpose of this study is to investigate the individual and contextual determinants of the perceived quality (PQ) of the telemedicine and teleassistance (TMTA) services and the willingness to continue (WC) with them among patients with diabetes using TMTA during the COVID-19 pandemic in one large region of Italy (Emilia-Romagna).

**Methods:**

A structured survey was administered to patients with type 1 and 2 diabetes who used TMTA services during the first wave of the COVID-19 pandemic. The questionnaire was comprised of questions on TMTA service experience and participants’ socio-demographic characteristics. Multiple regression models investigated the independent factors associated with PQ (score 1-100) and WC (yes/no).

**Results:**

The final analysis included 569 patients with diabetes (54.7% female), with an average age of 58.1 years. TMTA services’ PQ and WC were high. A higher education (OR = 1.83; 95%CI 1.04, 3.31) and being unemployed (OR = 2.57; 95%CI 1.17, 6.02) were factors associated with an increased WC. Older age was negatively related to PQ (b = −3.6; 95%CI −6.8, −0.29). Perceived support from TMTA service was positively associated with PQ (b = 10.1; 95%CI 5.1, 15) and WC (OR = 2.03; 95%CI 1.07, 3.85). Perceived increase in disease self-management was positively associated with PQ (b = 5.3; 95%CI 0.24, 10) and WC (OR = 7.11; 95%CI 4.04, 12.8).

**Conclusions:**

Our study identified several determinants of PQ and WC. These socio-demographic and patient-perception related factors should be considered in the implementation of care pathways integrating in-person visits with TMTA services.

**Key messages:**

• Socio-demographic factors play a crucial role in TMTA acceptance and should be taken into due consideration when implementing health pathways integrating in-person visits with TMTA services.

• Health workers should always try to improve patients’ self-management skills and should always make patients feel supported. This is also true in the digital health era.

